# Composite Nafion-CaTiO_3-δ_ Membranes as Electrolyte Component for PEM Fuel Cells

**DOI:** 10.3390/polym12092019

**Published:** 2020-09-04

**Authors:** Lucia Mazzapioda, Carmelo Lo Vecchio, Olesia Danyliv, Vincenzo Baglio, Anna Martinelli, Maria Assunta Navarra

**Affiliations:** 1 Department of Chemistry, Sapienza University of Rome, P.le Aldo Moro 5, 00185 Rome, Italy; lucia.mazzapioda@uniroma1.it; 2 CNR-ITAE, Istituto di Tecnologie Avanzate per l’Energia “Nicola Giordano”, Via Salita S. Lucia 5, 98126 Messina, Italy; lovecchio@itae.cnr.it (C.L.V.); baglio@itae.cnr.it (V.B.); 3 Department of Chemistry and Chemical Engineering, Chalmers University of Technology, 41296 Gothenburg, Sweden; olesia@chalmers.se (O.D.); anna.martinelli@chalmers.se (A.M.)

**Keywords:** composite polymer electrolytes, CaTiO_3−δ_ additive, oxygen vacancies

## Abstract

Manufacturing new electrolytes with high ionic conductivity has been a crucial challenge in the development and large-scale distribution of fuel cell devices. In this work, we present two Nafion composite membranes containing a non-stoichiometric calcium titanate perovskite (CaTiO_3−δ_) as a filler. These membranes are proposed as a proton exchange electrolyte for Polymer Electrolyte Membrane (PEM) fuel cell devices. More precisely, two different perovskite concentrations of 5 wt% and 10 wt%, with respect to Nafion, are considered. The structural, morphological, and chemical properties of the composite membranes are studied, revealing an inhomogeneous distribution of the filler within the polymer matrix. Direct methanol fuel cell (DMFC) tests, at 110 °C and 2 M methanol concentration, were also performed. It was observed that the membrane containing 5 wt% of the additive allows the highest cell performance in comparison to the other samples, with a maximum power density of about 70 mW cm^−2^ at 200 mA cm^−2^. Consequently, the ability of the perovskite structure to support proton carriers is here confirmed, suggesting an interesting strategy to obtain successful materials for electrochemical devices.

## 1. Introduction

For sustainable economic growth and environment protection, energy delivered from renewable sources is indispensable. In this field, fuel cell technologies are considered to be promising solution for a future clean energy environment. Among the different types of fuel cells, low temperature polymer electrolyte membrane fuel cells (PEMFCs), including direct methanol fuel cells (DMFCs), offer several advantages compared to other systems in terms of low emission of pollutants, high energy conversion and efficiency. However, these devices present two major drawbacks, namely their high cost and low durability, that must be solved for a large-scale application and commercialization [1,2]. 

In a typical low temperature PEMFC design, the perfluorosulfonic acid polymers are the state-of-the-art for solid electrolytes [3]. The most used polymer for these devices is Nafion®, manufactured by DuPont, due to beneficial features, such as high proton conductivity under fully hydrated conditions, suitable mechanical properties, high chemical and electrochemical stability, low fuel permeability, and electronic insulation [4,5]. In general, the proton conductivity of these membranes is approximately ~10^−1^ Scm^−1^ at room temperature and under fully hydrated conditions. Consequently, an adequate humidification of the Nafion membrane is necessary to obtain a good protonic conductivity of the electrolyte. However, in a fuel cell device under operation, the presence of an excess of water can prevent reactant diffusion to the catalysts at the electrode sides increasing the mass-transfer overpotential. The electrodes, in this case, are considered to be "flooded” and the performance of the cell drops drastically. Furthermore, increasing water content leads to an increased swelling of the polymer making it potentially water soluble and worsening the contact at the electrode sides. Because of problems with water management associated to the polymer, a PEM fuel cell is forced to operate at temperatures less than 100 °C. 

Several approaches have been considered for reducing the drawbacks associated with water management, based on the synthesis of alternatives membranes that are stable and high proton-conductive at higher temperatures. Many promising polymers based on aromatic thermoplastics, such as poly(aryl ether ketone)s (PAEKs), poly(ether sulfone) (PES), polybenzimidazole (PBI), and related materials, are studied as a replacement for Nafion membranes thanks to their features, such as high chemical and thermo-oxidative stability, good mechanical properties, and low cost [6]. In addition to this strategy, another approach consists of incorporating functional additives, such as Al_2_O_3_, TiO_2_, or sulfated metal oxides, within the Nafion matrix developing hybrid inorganic-organic polymer electrolyte membranes. These additives improve both the hydration state and proton conductivity of the membranes, decrease the cross-over of the gases during fuel cell operations and are particularly effective in PEMFCs at low RH and high temperature [7,8,9,10].

According to the most recent literature, inorganic perovskite-type oxides are becoming more and more fascinating nanomaterials for wide applications in catalysis, fuel cells, and other electrochemical devices thanks to their tunable properties and their intrinsic ionic and electrical conductivity, as well as their catalytic activity [11,12]. Compared to other types of metal oxides, the most appealing feature of perovskite oxides is their compositional and structural flexibility. Indeed, the perovskite design ABO_3_ allows to accommodate several doping agents, such as metal transition elements, in the A-site and/or B-site, providing oxygen vacancies in the lattice of the oxide [13]. The presence of the oxygen vacancies in the structure can play a key role as active sites for the dissociative absorption of water, which improves the hydrophilicity of the oxide by the protonation of the lattice oxygen ions. This phenomenon seems to be predominant in perovskite-type oxides due to the low formation enthalpies of oxygen ions because of their low bond strengths and strong relaxation effects. Furthermore, a distortion of the crystal lattice can influence the energetic properties of the oxygen ions in terms of different binding energies of protons [14]. Consequently, local structural and chemical perturbations induce water trapping effects and lattice relaxations around oxygen ion vacancies etc., which can significantly affect the proton mobility and conductivity.

Li et al. have studied by periodic DFT + U calculations the hydrogen transport behavior on the defect-free and defective LaMO_3_ surfaces (M = Cr, Mn, and Fe). In the presence of oxygen vacancies, the vehicular mechanism in which hydrogen moves together with the underlying oxygen would dominate on LaMnO_3_ and LaFeO_3_, whereas on the defective LaCrO_3_ the Grotthuss mechanism prevails [15]. According to Kreuer [16], water from the atmosphere dissociates into hydroxide ions and protons; the hydroxide ion fills the oxygen vacancy, and the proton forms a covalent bond with the lattice oxygen. This process can be written with the following equations using the Kröger–Vink notation:

H_2_O(g) + Vo^••^ + O_ox_ ⇌ 2 OH^•^

2 OH^•^ + ½ O_2_ ⇌ H_2_O(g) + 2h^•^ + 2O_ox_

2h^•^ + O_ox_ ⇌ Vo^••^ + ½ O_2_

where Vo^••^ represents the oxygen vacancy, O_ox_ is the oxygen in a regular crystal lattice site, OH^•^ is the protonic defect, and h^•^ is an electronic hole. Considering the formation of protonic defects as an amphoteric reaction, the oxide material can simultaneously act as an acid (absorption of hydroxide ion by oxygen ion vacancy) and as a base (protonation of lattice oxygen ions) [17]. The proton migration pathways are mainly characterized by lower activation barriers within solid oxides as compared to oxygen ions because of their smaller mass, lower radius, and absence of electron cloud. In a crystal lattice, protons are located close to their crystallographic position because of electrostatic attraction and can rotate and migrate between adjacent anions through the Grotthuss mechanism [18]. Consequently, in proton-conducting fuel cells, perovskite-type transition-metal oxides can be used as proton-conducting electrolytes given their high mobility of protonic defects.

In a previous work, we have used a calcium titanate perovskite (CaTiO_3−δ_, CTO) as a water-retention and reinforcing additive in low-humidity Nafion membranes obtaining, for the composite sample with a low concentration of filler, an improved protonic conductivity [19]. With the aim to better understand the behavior of the additive in the composite Nafion membranes, the present work is focused on new results obtained from measurements of water uptake, methanol crossover, and ion exchange capacity, as well as from small- and wide-angle x-ray scattering (SAXS and WAXS), vibrational spectroscopy (Raman and infrared), and high resolution field emission scanning electron microscopy (HR-FESEM).

## 2. Materials and Methods

A non-stoichiometric calcium titanate (CaTiO_3-δ_, CTO) perovskite was prepared by a solvo-thermal procedure as reported in our previous papers [19,20], using a Pluronic F127 both as a structure directing agent and as a reducing component to obtain oxygen vacancies in the lattice of the perovskite. This procedure results in an orthorhombic CaTiO_3-δ_ perovskitewith an average crystallite size of about 145 nm. The specific surface area of the particles, determined by the Brunauer–Emmett–Teller (BET) method, was 6.6 ± 0.5 m^2^ g^−1^ and an amount of oxygen vacancies corresponding to δ~0.025 was obtained [20].

All Nafion membranes were prepared by a solvent casting procedure [21]. The hydro-alcoholic solvents of a 5 wt.% Nafion ionomer solution (E.W. 1100, Ion Power Inc, München Germany) were evaporated and replaced with N,N-dimethylacetamide (> 99.5%, Sigma Aldrich, St. Louis, MO, USA) at 80 °C. Subsequently, the desired amount of CaTiO_3-δ_, to obtain weight ratios of 5% and 10%, was added to the mixture which was subsequently cast into a glass Petri dish and dried at 80 °C overnight. A plain, filler-free Nafion membrane was also prepared and used as internal benchmark. The samples will be referred to as M5, M10 and N, respectively. The thickness of all samples was measured in dry state, after removing them from the Petri dish and hot pressing (at 50 atm, 175 °C, for 15 min), resulting to be in the range of 90–110 µm. All as-formed membranes were finally pre-treated and purified in boiling 3 wt.% hydrogen peroxide (H_2_O_2_, 34.5%–36.5%, Sigma Aldrich, St. Louis, MO, USA), H_2_SO_4_ (0.5 M) and distilled water.

Composite membranes were evaluated in term of Ion Exchange Capacity (IEC) and Water Uptake (W.U.), which are important parameters since they provide a direct measure of the number of available protons and of the hydration level, respectively. The IEC was evaluated by a titration method. All dry membranes were immersed in a NaCl aqueous solution and the exchanged protons were neutralized with a standard solution of NaOH (0.1 M) [22]. The IEC error was estimated as the standard deviation from three different measurements. The total WU was evaluated at room temperature by a gravimetric method according to the following equation:

(1) $$W.U.=\frac{\left(W_{\mathrm{wet}}-W_{\mathrm{dry}}\right)}{W_{\mathrm{dry}}}*100\%$$

where *W*_wet_ is the weight of fully hydrated membranes, obtained by equilibrating the samples in a sealed container in the presence of water for two weeks, and *W*_dry_ is the weight of dry membranes measured after a night at 80 °C under vacuum. According to the literature [23], the above-mentioned properties can be used to estimate the value of λ, a parameter that allows to define the number of water molecules for each sulfuric acid group.

Small-angle X-ray scattering combined with wide-angle X-ray scattering (SAXS-WAXS) measurement have been used to understand the interactions between the filler and the Nafion matrix after hydration. In this case as well, the humidified membranes were obtained by equilibrating the samples in a close container in the presence of water for 4 days. These measurements were performed using a Mat:Nordic instrument from SAXSLAB/Xenocs. The instrument was equipped with a micro-focus Cu X-ray source and a Dectris Pilatus 300K R detector. The entire beam path was evacuated to 0.2 mbar before each measurement to minimize air scattering. The membranes were sandwiched between two mica windows in a holder suitable for solid self-standing films. The SAXSGui software was used for processing the data and the correlation distance between scattering objects was calculated using Bragg’s diffraction law *d* = 2π/*q*.

Vibrational spectroscopy studies were carried out by Attenuated Total Reflectance - Fourier Transform Infrared (ATR-FTIR) and Raman spectroscopy, to examine molecular interactions and chemical composition of all the membranes. Raman spectra were collected with an In-Via Reflex spectrometer from Renishaw, while the analysis of the spectra was performed using the WIRE 5.0 software. ATR-FTIR spectra were collected with a PerkinElmer 2000 FT-IR spectrometer in the attenuated total reflection mode using a ZnSe crystal. The spectral resolution was set to 1 cm^−1^ recording 64 scans for each sample at ambient temperature.

The morphology of the Nafion membranes was evaluated by high-resolution field emission scanning electron microscopy (HR-FESEM), using an Auriga Zeiss instrument at the Interdepartmental Research Center on Nanotechnologies applied to Engineering (CNIS) of Sapienza University of Rome.

Methanol crossover measurements were carried out electrochemically in a typical fuel cell configuration [24]. Membrane-electrode assemblies (MEAs) were obtained by hot-pressing the Pt-based electrodes onto the prepared composite or filler-free membranes. Linear sweep voltammetry (LSV) was performed in the voltage range of 0–0.9 V with a scan rate of 2 mV s^−1^. A 2 M MeOH solution (3 mL min^−1^) was fed to one side of the cell used as the counter/reference electrode, while N_2_ (100 mL min^−1^) was supplied to the other compartment (hence operating as the working electrode). Methanol crossing the membrane is oxidized at the working electrode generating a positive current, which reaches a plateau when all methanol is converted to CO_2_ under steady state conditions [25,26]. The same MEAs were used for the direct methanol fuel cell tests. The anode was made by mixing a 60% Pt-Ru/C (Alfa Aesar) as the catalyst and a 5% Nafion® (Ion Power) solution (33 wt.% with respect to the catalyst amount) as the ionomer; whereas, for the cathodic catalytic layer a mixture of 20% Pt/C (E-TEK) and Nafion® ionomer in a ratio 67:33 as for the anode was used. They were deposited by a doctor blade technique onto the diffusion backing layers above reported with a Pt loading of 1.5 mg cm^−2^ at the anode and 0.5 mg cm^−2^ at the cathode. The MEAs were tested in a 5 cm^2^ single cell fixture (Fuel Cell Tech., Inc.).

## 3. Results and Discussion

The ion exchange capacity of the Nafion sample approaches its theoretical value of 9 × 10^−4^ eqg^−1^ considering that the Nafion polymer used for this research has an equivalent weight of 1100 geq^−1^. None of the composite membranes shows a dramatic decrease of the IEC values compared to Nafion. Typically, composite systems show a significant decrease of their IEC due to an increase of density in the membrane because of the presence of inorganic particles which also can lead to fewer available exchangeable protons. In Table 1, the measured values of IEC, W.U. and λ are reported, revealing comparable properties for all samples.

**Figure 1:**
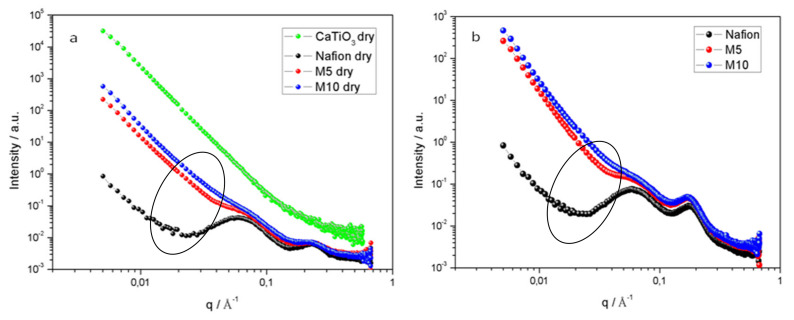
SAXS and WAXS patterns of all the Nafion membranes here investigated under dry (**a**) and humidified (**b**) conditions. The pattern of CTO dry powder is also reported for comparison purpose.

Figure 1 shows the scattering intensity I(q) obtained by SAXS and WAXS measurements for all the membranes under investigation at both dry and humidified conditions. In Figure 1a, the scattering profile of the neat and dry perovskite calcium titanate is shown for comparative purposes (green trace) and to justify the slope observed at lower q values for the composite Nafion membranes. 

In these plots, two main peaks emerge which give information on the shape and size of the scattering objects in the Nafion membranes. The first peak, also called the matrix knee, corresponds to the fluorocarbon polymer crystallites randomly distributed in the amorphous polymer matrix. Here, it is observed at q values close to 0.06 Å^−1^ and its intensity depends on the polymer’s degree of crystallinity [27].

The second peak, also known as the ionomer peak, is here observed in the q range 0.2–0.3 Å^−1^ and arises from the local ordering of the ionic domains within the polymer. From the position and intensity of the ionomer peak, it is possible to achieve information about the hydration degree of the Nafion sample, since it is related to the periodicity of the water channels within the membrane. As a note, the Nafion membrane’s structure can be described as rod-like ionic domains that expand radially upon hydration. This model is considered a more appropriate description than the Gierke’s clustering model [28], which considers spherical ionic hydrated clusters connected by narrower channels only 1 nm wide. 

In order to evaluate the hydration state of the Nafion membranes, the correlation distance *d* was calculated from Bragg’s diffraction law *d* = 2π/*q*, where *q* is the center of the ionomer scattering peak. In Table 2, the values of *d* and *q* are reported for all samples at both investigated conditions.

As it can be seen from Table 2, all Nafion membranes (undoped and doped) in the dry state display scattering peaks at equivalent positions and hence comparable d values. However, when the membranes are subjected to hydration, the position of the ionomer peak shifts systematically to lower q values, which is particularly evident for the two composite membranes. These values reflect an increased size of the ionic domain as a result of the absorbed water. Among the investigated samples, M10 shows the highest d value indicating a higher degree of hydration and confuting the W.U. values obtained by weight. Anyhow, our previous results based on calorimetric measurements and dielectric spectroscopy [19] revealed that M5 sample has displayed the highest water affinity and conductivity, suggesting that an intermediate filler concentration (i.e., 5 wt.%) could be the best compromise. It can be assumed that the presence of oxygen vacancies in the perovskite structure guarantees the water trapping but a high dose of CTO (i.e., 10 wt.%) in the Nafion matrix can cause a lack of relative interactions between the polymer, additive and water to ensure the mobility of the protons. It’s worth noticing from the trends in Figure 1 that the matrix knee peak is still visible in M5 sample, whereas it almost disappears in M10.

**Figure 2:**
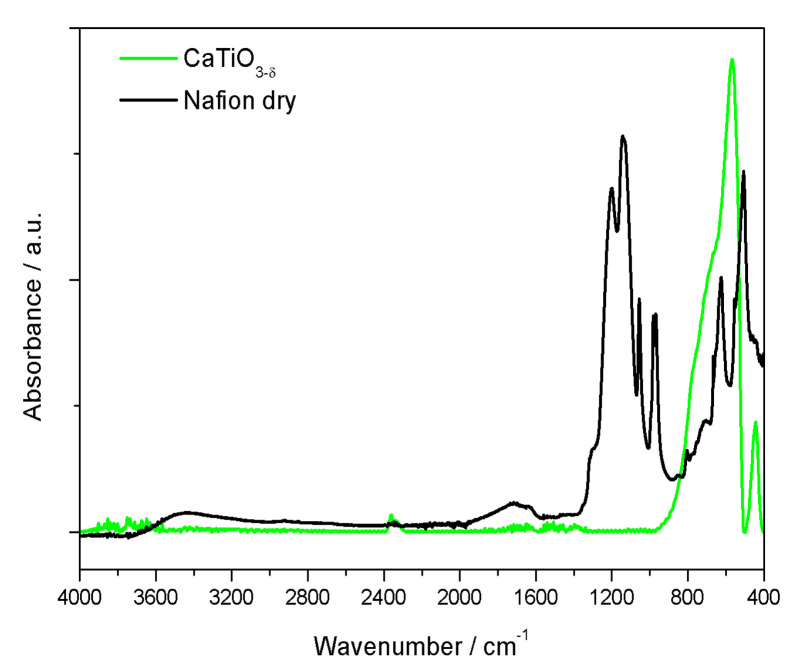
Infrared spectra of a Nafion membrane and CTO powder.

Figure 2 shows the FTIR spectra of a Nafion membrane in the dry state and that of the neat calcium titanate perovskite. In the FTIR spectra of Figure 2 the following vibrational modes can be distinguished: the hydrophobic fluorocarbon chains of Nafion (1300–1000 cm^−1^) and the perfluoroetheral side chains of Nafion (1000–940 cm^−1^), as well as the Ti-O stretching vibration and the Ti-O-Ti bridging stretching modes of the inorganic component (430–560 cm^−1^). These spectral features are in agreement with those observed in the IR spectra of CaTiO_3_ already reported by other authors [29]. In the low wavenumber range, the peaks arising from the inorganic additive overlap with those of Nafion. The assignment of the main peaks from Nafion is given in Table 3 [30]. The ʋ_s_ (SO_3_^-^) mode at 1059 cm^−1^ was chosen for the intensity normalization of all IR spectra.

Moreover, the spectrum of the Nafion membrane exhibits broad features just above 1600 cm^−1^ and at about 3500 cm^−1^ that are related to the bending and stretching modes of water still present despite the drying treatment at 80 °C prior to measurement. Interestingly, for the composite membranes, the IR spectra show some differences depending on the side of the membrane analyzed.

**Figure 3:**
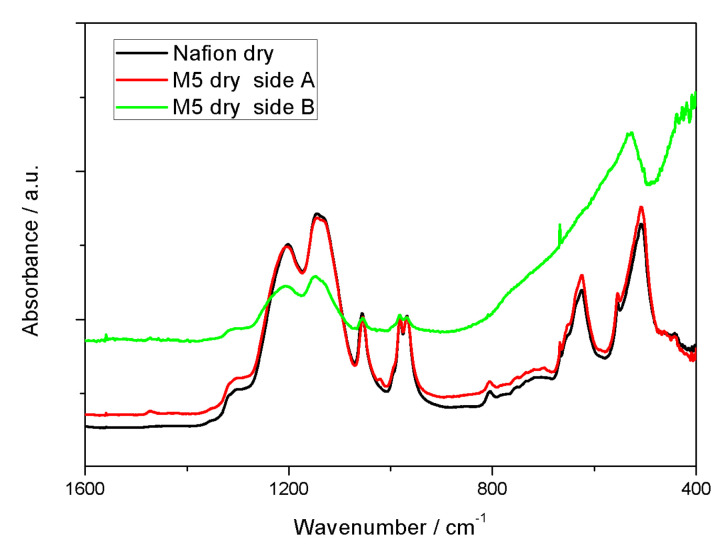
FTIR Spectra of a dry Nafion membrane (black) and a dry M5 membrane when facing side A (red) or side B (green) towards the ATR crystal.

As shown in Figure 3 for the case of sample M5 (the composite membraneM10 displays the same behavior and is not here reported), strong differences are observed when analyzing the two opposite sides of the membrane: the peaks assigned to Nafion appear more intense on one side (here called side A) whereas the peaks related to the perovskite are more emphasized on the opposite side (here called side B). As already mentioned in the literature, during the solvent casting process, the filler can develop a concentration profile [31], making the Nafion membrane not completely uniform in terms of dispersion of the additive, especially in the region closest to the surface. 

**Figure 4:**
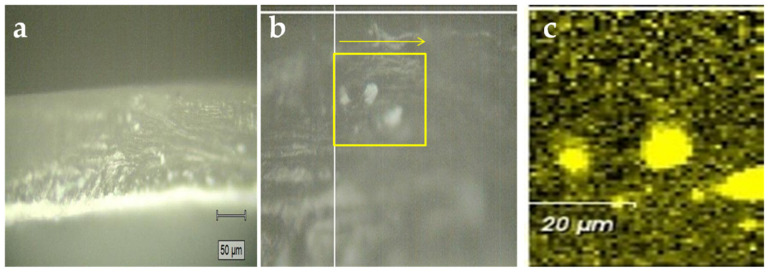
(**a**) Microscope images of a M5 membrane sample taken from side-view obtained during the Raman analysis; (**b**) area, within the yellow box, selected for mapping Raman analysis; (**c**) Nafion (black zone) and CTO (yellow zone) distribution in the selected area.

**Figure 5:**
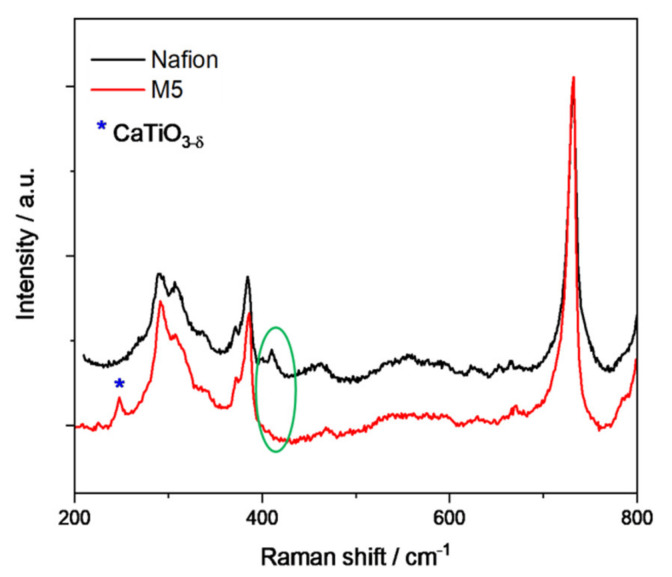
Raman spectra of a M5 membrane compared to Nafion membrane.

The presence of two different sides in the Nafion membrane was also confirmed by Raman spectroscopy, by means of microscope images and 2D mapping over cross sections of the M5 sample, as reported in Figure 4 and Figure 5.

Raman spectra were collected over selected areas with step increases of1 micrometers. Then, a color-coded image could be obtained by mapping the intensity of selected peaks, in this case the peak at 732 cm^−1^ representative for the presence of Nafion and the peak at 250 cm^−1^ representative of the presence of CaTiO_3_. As evident from Figure 4, in the composite membranes some micrometers large CaTiO_3_ aggregates tend to form in the bulk of the membrane, while a distinct CaTiO_3_ layer is formed close to one of the surfaces.

The Raman spectrum of M5 sample, obtained as an average of the spectra recorded in the selected area, is shown in Figure 5 and it was normalized with respect to the band peaking at 732 cm^−1^, which corresponds to the ʋ(CF_2_) mode of Nafion as reported in Table 4. As discussed for the IR spectra, also for the Raman spectra all the peaks due to the Calcium Titanate perovskite overlap with those of Nafion, except for a weak one at ca. 250 cm^−1^ assigned to the O–Ti–O bending mode [32]. The major characteristic vibrational bands of the polymer are assigned as given in Table 4 [33]. However, some important differences between plain Nafion and composite M5 membrane have been noticed: that is the absence in the composite membrane of bands at about 398 and 410 cm^−1^ related to CF_2_ stretching vibration (marked with a green circle) which is the signature of an important phase rearrangement of the calcium titanate component incorporated into the Nafion membrane.

The functionality of the membranes here proposed were tested with respect to direct methanol fuel cell (DMFC) applications. In DMFC, methanol crossover is a critical issue which is responsible of cathode catalyst poisoning causing about a 30% performance reduction in terms of fuel cell efficiency [34]. Nafion possesses high methanol permeability because of its hydrophilic channels but hosting suitable additives within these channels would significantly reduce the crossover. 

**Figure 6:**
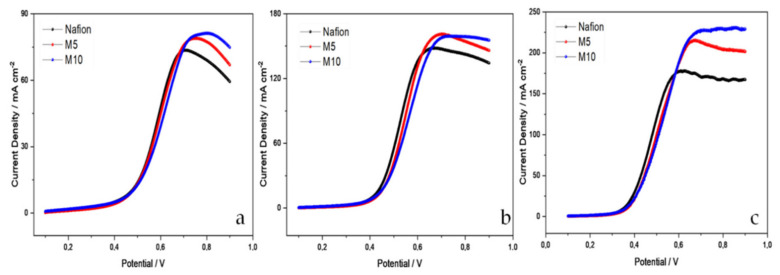
Methanol crossover current densities at (**a**) 30 °C, (**b**) 60 °C and (**c**) 90 °C for the composite membranes compared with the filler-free Nafion in the presence of 2M methanol.

In accordance to the literature, in the composite approach, the extent of methanol crossover largely depends on the distribution of fillers and their effective interaction with the polymer matrix [35]. The uniform distribution of fillers in Nafion membranes reduces the size of channels that are available for methanol passage, whereas particles agglomeration has a negative impact on methanol crossover. Figure 6 shows the methanol crossover behavior for the two composite membranes compared with a filler-free Nafion membrane of similar thickness in a wide range of temperatures (from 30 to 90 °C) feeding a 2 M methanol solution to one side of the cell used as counter/reference electrode.

Methanol crossing the membrane is oxidized at the other electrode (working electrode) generating a positive current, which reaches a plateau when all methanol is converted to CO_2_. Unfortunately, as observed in Figure 6, it appears that methanol crossover slightly increases after modification of the Nafion membrane with CaTiO_3-δ_ particles. The value of current density represents the amount of methanol passing through the membrane; it increases with the temperature, due to a higher mobility of methanol. At 60 °C, the value of the so-called crossover current density for all membranes is in the range of 130–160 mA cm^−2^, lower than the state-of-the-art Nafion 115 (125 µm in thickness), which showed 195 mA cm^−2^ crossover current in a previous work [36].

At 90 °C a further increase of methanol crossover has been reported for Nafion 115 membrane (380 mA cm^−2^) [37]; whereas, the filler-free and composite membranes with CTO present lower values, in the range 180−220 mA cm^−2^, with the filler-free having the lowest methanol crossover.

**Figure 7:**
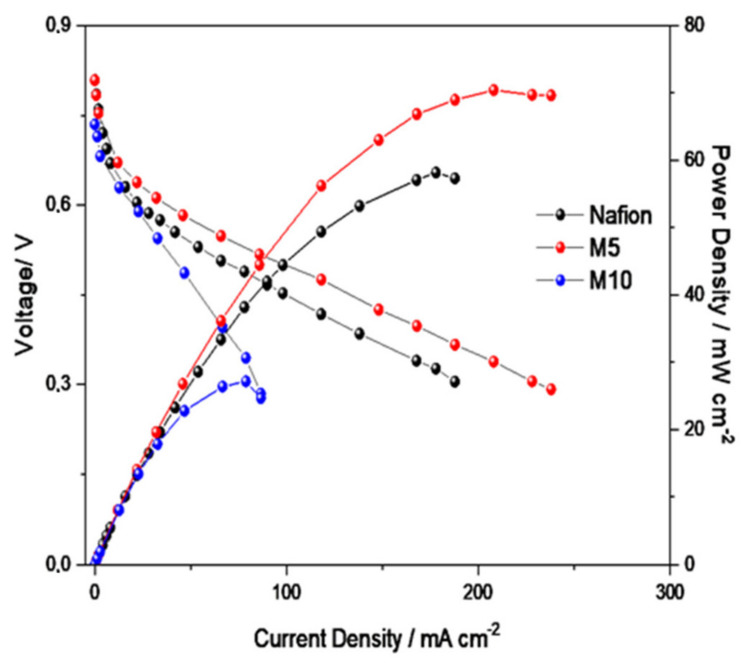
Polarization and power density curves at 110 °C and 2 M methanol concentration at the anode for the DMFCs based on the different membranes, filler-free Nafion, Nafion with 5 wt.% CTO (M5) and Nafion with 10 wt.% CTO (M10).

Since the composite membranes were designed to allow operation of a DMFC (or a PEFC) at temperatures higher than 90–100 °C [38,39], reducing the drawback of membrane dehydration at those temperatures with consequent loss of conductivity. The influence of the CTO filler on the electrochemical behavior of the membrane in DMFC at 110 °C has been evaluated, and the results are reported in Figure 7.

As observed, the composite membrane with 5% CTO additive provided the highest performance in the fuel cell among the different membranes, confirming the fact that at temperatures higher than 100 °C the filler helps the proton conduction mechanism in particular in the presence of a lower water content and also enhances the mechanical properties and the stability of the Nafion membrane, as reported in a previous paper [19]. Several researches confirm that good mechanical properties are crucial to obtain better fuel cells performances especially at high temperature [40,41]. Consequently, the role of calcium titanate perovskite as a water retention and reinforcing additive has been confirmed.

The presence of the oxygen vacancies in the structure of CTO can play a key role as active sites for the absorption of water. Furthermore, a distortion of the crystal lattice can influence the energetic properties of the oxygen ions in terms of different binding energies of protons [14], with a consequent increase of the proton mobility and conductivity. Unfortunately, a large presence of CTO (10%) in the polymer is not beneficial, probably due to a large presence of agglomerates, and thus defects in the membrane morphology (presence of micro-holes) which cause an increase of methanol crossover and a decrease of performance.

**Figure 8:**
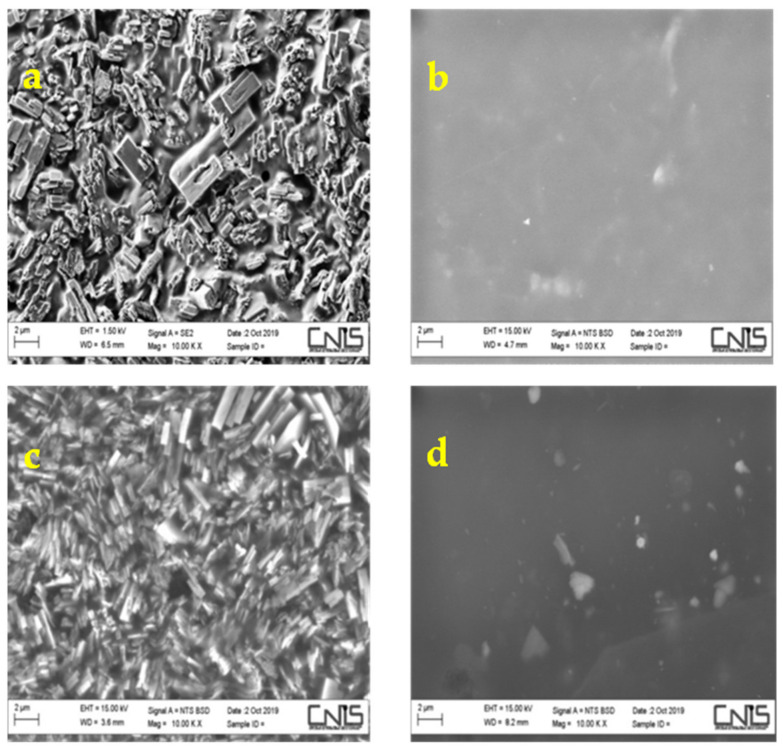
Top: SEM images of both M5 sides, one rich in perovskite (**a**) the other poor (**b**). Bottom: SEM images of both M10 sides, one rich in perovskite (**c**) the other poor (**d**).

This could be understood better from the SEM images (Figure 8), revealing a surface morphology of composite Nafion membranes where the additive particles are not uniformly distributed, giving rise to two different sides as also demonstrated by Raman spectroscopy.

In particular, comparing the two composite membranes on the side rich in perovskite, it is remarkable that in M5 the mixing of the perovskite with Nafion uniformly occurs on the surface of the membrane whereas in M10 sample the presence of the perovskite is so pronounced to hide completely the Nafion component. 

At this point it is clear the existence of a critical filler concentration that influences the morphology of the composite membrane. Furthermore, as reported in previous studies, an excessive additive begins to form agglomerates in the composite membrane, the hydrophobic polymer backbones will occur around the hydrophilic ion-cluster for methanol permeation, increasing the whole permeability [42].

In our case, the bad performance in terms of methanol crossover can be further explained considering another characteristic aspect of the calcium titanate additive, i.e., the presence of oxygen vacancies in the lattice of the perovskite. The oxygen defects have been considered as a strategy to improve the number of active sites able to absorb oxygen-containing ligands, such as methanol molecules. 

Yang et al. [43] reported a study about an ultra-thin nickel oxide rich in oxygen vacancies, demonstrating that the presence of these oxygen defects can enhance the methanol oxidation reaction (MOR) performance. Furthermore, as reported in our previous work [20], it was demonstrated that CTO could improve the methanol oxidation if it is used as additive to Pt/C catalyst, confirming a promoting effect of the perovskite for this reaction.

Consequently, the capability of oxygen vacancies to interact with methanol molecules can support the methanol permeability through the Nafion membranes, justifying the results obtained. Overall, in view of DMFC applications, the non-uniform additive distribution can be overcome by reducing the inorganic particles size and/or properly modifying the membrane preparation to obtain thinner and uniform CTO/Nafion composites (e.g., by moving from a solvent-casting procedure to a spray coating or thin film deposition methods). In contrast, as an interesting original approach, the additive-rich side can be conveniently adopted at the cathode interface of a DMFC to increase reactivity towards ORR.

## 4. Conclusions

In the present work, two composite membranes based on a Nafion polymer matrix incorporating a CaTiO_3-δ_ additive have been proposed and characterized. A detailed analysis in terms of structure and morphology was performed. Applicability in DMFCs was also tested. An increase of methanol crossover was observed in the composite membranes, most likely due to both the non-uniform distribution of the filler within the polymer and the presence of oxygen vacancies in the lattice of the perovskite, that can promote the methanol permeability through the Nafion membrane. From both ATR and Raman vibrational spectroscopy, as well as from SEM images, two sides with different morphology and filler concentration have been identified in the composite systems.

The issues of inhomogeneous dispersion and non-optimized filler-to-polymer interactions appear particularly critical in this work and are believed to prevent the beneficial, functional effect of oxygen vacancies in the perovskite structure. Moreover, a too high filler concentration could obstruct the ionic channels and impede ionic motion, influencing the performance of the composite membranes during fuel cell operations.

Anyhow, the reported results indicate that composite membranes, obtained by adding a suitable concentration of non-stoichiometric calcium titanate in a Nafion matrix, display interesting properties that ought to be considered for fuel cell applications. Indeed, improved performances, in terms of current and power delivered, were observed when using a 5 wt% CTO-added Nafion in a DMFC operating at 110 °C.

## Figures and Tables

**Table 1:** Values of WU, IEC and λ of membranes equilibrated at 100% RH.

Sample	IEC (meq g^−1^)	W.U. (%)	λ (H_2_O/SO_4_^2−^)
N	0.83 ± 0.1	18.9	12.7
M5	0.80 ± 0.03	18.1	12.6
M10	0.90 ± 0.04	17.4	12.2

**Table 2:** Values of q and d estimated for all the samples at dry and humidified conditions.

	Dry		Humid	
Sample	q/Å^−1^	d/Å	q/Å^−1^	d/Å
N	0.23	27.08	0.20	31.89
M5	0.23	26.97	0.18	35.90
M10	0.23	27.08	0.17	37.85

**Table 3:** Main vibrational modes of Nafion.

Frequency/cm^−1^	Assignment [30]
1300	ʋ_s_ (C–C)
1206	ʋ_as_ (CF_2_)
1142	ʋ_s_ (CF_2_)
1059	ʋ_s_ (SO_3_^−^)
982	ʋ_s_ (C–O–C)
969	ʋ_s_ (C–O–C)

**Table 4:** Assignment of major Raman bands of Nafion.

Raman Shift/cm^−1^	Assignment [33]
292, 310 307, 385, 390, 574	ʋ (CF_2_)
732, 741	ʋ (CF_2_)

## References

[1] Vielstich W., Lamm A., Gasteiger H. (2003). Handbook of Fuel Cells—Fundamentals, Technology and Applications.

[2] Aricò A.S., Baglio V., Antonucci V., Liu H., Zhang J. (2009). Direct methanol fuel cells: Hystory, status and perspectives. Electrocatalysis of Direct Methanol Fuel Cells: From Fundamentals to Applications.

[3] Kundu S., Simon L.C., Flowler M., Grot S. (2005). Mechanical Properties of Nafion™ Electrolyte Membranes under Hydrated Conditions. Polymer.

[4] Siracusano S., Oldani C., Navarra M.A., Tonella S., Mazzapioda L., Briguglio N., Aricò A.S. (2019). Chemically stabilised extruded and recast short side chain Aquivion^®^ proton exchange membranes for high current density operation in water electrolysis. J. Membr. Sci..

[5] Ahmad M.I., Zaidi S.M.J., Rahman S.U. (2006). Proton conductivity and characterization of novel composite membranes for medium-temperature fuel cells. Desalination.

[6] Li L., Zhang J., Wang Y. (2003). Sulfonated poly (ether ether ketone) membranes for direct methanol fuel cell. J. Membr. Sci..

[7] Branchi M., Sgambetterra M., Pettiti I., Panero S., Navarra M.A. (2015). Functionalized Al_2_O_3_ particles as additives in proton-conducting polymer electrolyte membranes for fuel cell applications. Int. J. Hydrog. Energy.

[8] Scipioni R., Gazzoli D., Teocoli F., Palumbo O., Paolone A., Ibris N., Brutti S., Navarra M.A. (2014). Preparation and characterization of nanocomposite polymer membranes containing functionalized SnO_2_ additives. Membranes.

[9] Nicotera I., Kosma V., Simari C., Ranieri G.A., Sgambetterra M., Panero S., Navarra M.A. (2015). An NMR study on the molecular dynamic and exchange effects in composite Nafion/sulfated titania membranes for PEMFCs. Int. J. Hydrog. Energy.

[10] Mazzapioda L., Panero S., Navarra M.A. (2019). Polymer electrolyte membranes based on Nafion and a superacidic inorganic additive for Fuel Cell applications. Polymers.

[11] Pena M.A., Fierro J.L.G. (2001). Chemical structures and performance of perovskite oxides. Chem. Rev..

[12] Suntivich J., Gasteiger H.A., Yabuuchi N., Nakanishi H., Goodenough J.B., Shao-Horn Y. (2011). Design principles for oxygen-reduction activity on perovskite oxide catalysts for fuel cells and metal-air batteries. Nat. Chem..

[13] Fabbri E., Mohamed R., Levecque P., Conrad O., Kötza R., Schmidt T.J. (2014). Unraveling the oxygen reduction reaction mechanism and activity of d-band perovskite electrocatalysts for low temperature alkaline fuel cells. ECS Trans..

[14] Stølen S., Bakkenw E., Mohn C.E. (2006). Oxygen-deficient perovskites: Linking structure, energetics and ion transport. Phys. Chem. Chem. Phys..

[15] Li Q., Yin Q., Zheng Y.S., Sui Z.J., Zhou X.G., Chen D., Zhu Y.A. (2019). Insights into Hydrogen Transport Behavior on Perovskite Surfaces: Transition from the Grotthuss Mechanism to the Vehicle Mechanism. Langmuir.

[16] Kruner K.D. (2003). Proton-conducting oxides. Annu. Rev. Mater. Res..

[17] Kreuer K.D., Adams S., Münch W., Fuchs A., Klock U., Maier J. (2001). Proton conducting alkaline earth zirconates and titanates for high drain electrochemical applications. Solid State Ionics.

[18] Pionke M., Mono T., Schweika W., Springer T., Schober H. (1997). Investigation of the hydrogen mobility in a mixed perovskiteBa [Ca_(1+x)/3_Nb_(2−x)/3_]O_3−x/2_ by quasielastic neutron scattering. Solid State Ionics.

[19] Mazzapioda L., Navarra M.A., Trequattrini F., Paolone A., Elamin K., Martinelli A., Palumbo O. (2019). Composite Nafion membranes with CaTiO_3-δ_ additive for possible applications in electrochemical devices. Membranes.

[20] Mazzapioda L., Lo Vecchio C., Paolone A., Aricò A.S., Baglio V., Navarra M.A. (2019). Enhancing oxygen reduction reaction catalytic activity using a sub-stoichiometric CaTiO_3−δ_ additive. ChemElectroChem.

[21] Siracusano S., Baglio V., Nicotera I., Mazzapioda L., Aricò A.S., Panero S., Navarra M.A. (2017). Sulfated titania as additive in Nafion membranes for water electrolysis applications. Int. J. Hydrog. Energy.

[22] Slade S.M., Ralph T.R., de Ponce León C., Campbell S.A., Walsh F.C. (2010). The Ionic Conductivity of a Nafion^®^ 1100 Series of Proton-exchange Membranes Re-cast from Butan-1-ol and Propan-2-ol. Fuel Cells.

[23] Neelakandan S., Kanagaraj P., Nagendran A., Rana D., Matsuura T., Muthumeenal A. (2015). Enhancing proton conduction of sulfonated poly (phenylene ether ether sulfone) membrane by charged surface modifying macromolecules for H_2_/O_2_ fuel cells. Renew. Energy.

[24] Simari C., Lo Vecchio C., Baglio V., Nicotera I. (2020). Sulfonated polyethersulfone/polyetheretherketone blend as high performing and cost-effective electrolyte membrane for direct methanol fuel cells. Renew. Energy.

[25] Qi Z., Kaufman A. (2002). Open circuit voltage and methanol crossover in DMFCs. J. Power Sources.

[26] Lufrano F., Baglio V., Di Blasi O., Staiti P., Antonucci V., Aricò A.S. (2012). Solid polymer electrolyte based on sulfonated polysulfone membranes and acidic silica for direct methanol fuel cells. Solid State Ionics.

[27] Fernandez Bordína S.P., Andrada H.E., Carrerasa A.C., Castellano G.E., Oliveira R.G., Galván Josa V.M. (2018). Nafion membrane channel structure studied by small-angle X-ray scattering and Monte Carlo simulations. Polymer.

[28] Deabate S., Gebel G., Huguet P., Morin A., Pourcelly G. (2012). 3in situ and operando determination of the water content distribution in proton conducting membranes for fuel cells: A critical review. Energy Environ. Sci..

[29] Wang Y., Niu C.G., Wang L., Yin Wang Y., Zhang X.G., Zeng G.M. (2016). Synthesis of fern-like Ag/AgCl/CaTiO_3_ plasmonic photocatalysts and their enhanced visible-light photocatalytic properties. RSC Adv..

[30] Singh R.K., Kunimatsu K., Miyatake K., Tsuneda T. (2016). Experimental and Theoretical Infrared Spectroscopic Study on Hydrated Nafion Membrane. Macromolecules.

[31] Suna C., Zlotorowicza A., Nawna G., Negro E., Bertasia F., Pagota G., Vezzù K., Pace G., Guarnierie M., Di Noto V. (2018). Nafion/(WO_3_)_x_] hybrid membranes for vanadium redox flow batteries. Solid State Ionics.

[32] Balachandran U., Eror N.G. (1982). Laser-induced Raman scattering in Calcium Titanate. Solid State Commun..

[33] Gruger A., Régis A., Schmatko T., Colomban P. (2001). Nanostructure of Nafion membranes at different states of hydration: An IR and Raman study. Vib. Spectrosc..

[34] Chen F., Mecheri B., D’Epifanio A., Traversa E., Licoccia S. (2010). Development of Nafion/Tin Oxide Composite MEA for DMFC Applications. Fuel Cells.

[35] Wang Y., Guimei H., Tian Z., Wang M., Liab J., Wang X. (2014). Nafion^®^/SiO_2_/m-BOT composite membranes for improved direct methanol fuel cell performance. RSC Adv..

[36] Aricò A.S., Sebastian D., Schuster M., Bauer B., D’Urso C., Lufrano F., Baglio V. (2015). Selectivity of Direct Methanol Fuel Cell Membranes. Membranes.

[37] Simari C., Enotiadis A., Lo Vecchio C., Baglio V., Coppola L., Nicotera I. (2020). Advances in hybrid composite membranes engineering for high-performance direct methanol fuel cells by alignment of 2D nanostructures and a dual-layer approach. J. Membr. Sci..

[38] Nicotera I., Simari C., Coppola L., Zygouri P., Gournis D., Brutti S., Minuto F.D., Aricò A.S., Sebastian D., Baglio V. (2014). Sulfonated Graphene Oxide Platelets in Nafion Nanocomposite Membrane: Advantages for Application in Direct Methanol Fuel Cells. J. Phys. Chem. C.

[39] Velayutham P., Sahu A., Parthasarathy S. (2017). A Nafion-Ceria composite membrane electrolyte for reduced methanol crossover in Direct Methanol Fuel Cells. Energies.

[40] Chongshan Y., Bangyun X., Qicheng L., Jingjing L., Libing Q., Yawei Z., Chunqing H. (2019). Lateral-aligned sulfonated carbon-nanotubes/Nafion composite membranes with high proton conductivity and improved mechanical properties. J. Membr. Sci..

[41] Kwangjin O., Osung K., Byungrak S., Dong H.L., Sangaraju S. (2019). Nafion-sulfonated silica composite membrane for proton exchange membrane fuel cells under operating low humidity condition. J. Membr. Sci..

[42] Yang W., Yang X., Jia J., Hou C., Gao H., Mao Y., Wang C., Lin J., Luo X. (2019). Oxygen vacancies confined in ultrathin nickel oxide nanosheets for enhanced electrocatalytic methanol oxidation. Appl. Catal. B Environ..

[43] Mazzapioda L., Lo Vecchio C., Aricò A.S., Navarra M.A., Baglio V. (2019). Performance Improvement in Direct Methanol Fuel Cells by Using CaTiO_3-δ_ Additive at the Cathode. Catalysts.

